# The complete mitochondrial genome of *Schoutedenia ralumensis* Rübsaamen, 1905 (Hemiptera: Aphididae: Greenideinae)

**DOI:** 10.1080/23802359.2020.1768938

**Published:** 2020-05-28

**Authors:** Jing Chen, Liyun Jiang, Xiaolu Zhang, Gexia Qiao

**Affiliations:** a Key Laboratory of Zoological Systematics and Evolution, Institute of Zoology, Chinese Academy of Sciences, Beijing, China; b College of Life Sciences, University of Chinese Academy of Sciences, Beijing, China

**Keywords:** Aphids, mitogenome, repeat region, phylogeny

## Abstract

We sequenced the complete mitochondrial genome of *Schoutedenia ralumensis*. The mitogenome is 16,051 bp long with an A + T content of 84.5%, including 13 protein-coding genes, 22 transfer RNA genes, 2 ribosomal RNA genes, a control region, and an aphid-specific repeat region located between *trnE* and *trnF*. All protein-coding genes are initiated by ATN and terminated with TAA or TAG except for *cox1* and *nad5*. All transfer RNAs display the typical clover-leaf secondary structure except for *trnS (AGN)*. The unique repeat region is 974 bp long, in which a 305-bp repeat unit repeats 3.19 times. The phylogenetic tree supports a sister relationship of *S. ralumensis* and *Greenidea psidii*.

*Schoutedenia ralumensis* Rübsaamen, 1905 is an aphid species widely distributed in southeastern Asia, India, Africa and Australia. It feeds on the plants of Euphorbiaceae, with a monoecious and partially holocyclic life cycle (Blackman and Eastop 2020). In this study, using Illumina sequencing, we characterize the complete mitochondrial genome of *S. ralumensis*, the first representative from the aphid tribe Schoutedeniini (Aphididae: Greenideinae). The aphid samples were collected on *Breynia fruticosa* from Mt. Wuzhi, Hainan, China (18.9045°N, 109.6820°E) and deposited in the National Zoological Museum of China, Institute of Zoology, Chinese Academy of Sciences, Beijing, China (NZMC no. 24309).

The *S. ralumensis* mitogenome is 16,051 bp long (GenBank accession number MT381994), which comprises 13 protein-coding genes, 22 transfer RNA genes (tRNAs), 2 ribosomal RNA genes (rRNAs), a control region, and a repeat region located between *trnE* and *trnF*. All 37 genes are arranged in the same order as the inferred ancestral arrangement of insects (Clary and Wolstenholme 1985). The overall nucleotide composition is 38.9% T, 9.8% C, 45.6% A and 5.7% G, with an A + T content of 84.5%. All protein-coding genes use the typical ATN start codons and TAA or TAG stop codons except for *cox1* and *nad5*, which end with a single T. The tRNA genes range from 62 to 73 bp in length. All tRNAs exhibit a typical clover-leaf secondary structure except for *trnS (AGN)*, which losses the dihydrouridine (DHU) arm. The *rrnL* and *rrnS* genes are 1267 and 767 bp in length, with an A + T content of 86.2% and 84.9%, respectively.

The control region located between *rrnS* and *trnI* is 542 bp long with an A + T content of 84.2%. The unique repeat region between *trnE* and *trnF*, which has been found in previously reported mitogenomes of Greenideinae species (i.e. *Greenidea psidii* van der Goot and *Greenidea ficicola* Takahashi) (Chen et al. 2019; Liu et al. 2020), is also present in the mitogenome of *S. ralumensis*. It is 974 bp in length with an A + T content of 85.5%. A 305-bp repeat unit repeats 3.19 times in this region.

Based on the whole mitochondrial genome sequences of *S. ralumensis* and 23 other aphid species, we constructed a maximum-likelihood phylogenetic tree of aphids using RAxML v8.2.10 (Stamatakis 2014). The Greenideinae was recovered as a monophyletic clade with strong support and *S. ralumensis* was firmly placed as a sister to *G. psidii* (Figure 1). The subfamilies Hormaphidinae, Calaphidinae and Aphidinae were all monophyletic, whereas the Eriosomatinae was polyphyletic.

**Figure 1.**
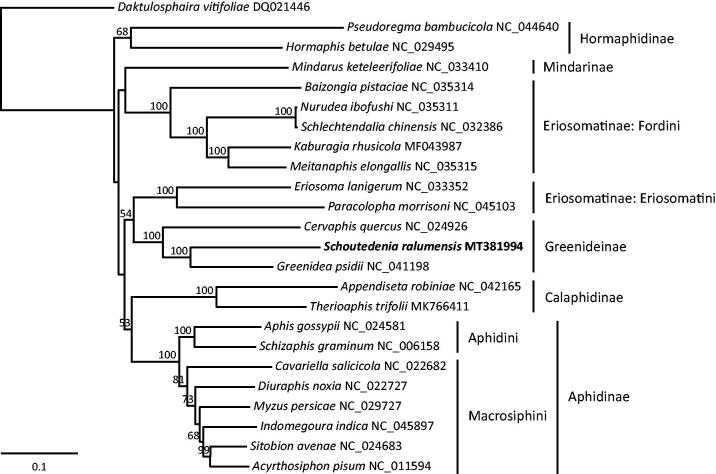
The maximum-likelihood tree inferred from the whole mitochondrial genomes of *Schoutedenia ralumensis* and 23 other aphids. Bootstrap values (>50%) are shown above the branches.

## Data Availability

The data that support the findings of this study are openly available in Dryad at https://doi.org/10.5061/dryad.dv41ns1vg.
